# Thirteen-Valent Pneumococcal Conjugate Vaccine–Induced Immunoglobulin G (IgG) Responses in Serum Associated With Serotype-Specific IgG in the Lung

**DOI:** 10.1093/infdis/jiab331

**Published:** 2021-06-22

**Authors:** Elena Mitsi, Daniella McLenaghan, Asia-Sophia Wolf, Scott Jones, Andrea M Collins, Angela D Hyder-Wright, David Goldblatt, Robert S Heyderman, Stephen B Gordon, Daniela M Ferreira

**Affiliations:** 1 Department of Clinical Sciences, Liverpool School of Tropical Medicine, Liverpool, United Kingdom; 2 National Institute for Health Research Global Health Mucosal Pathogens Research Unit, Division of Infection and Immunity, University College London, London, United Kingdom; 3 Institute of Child Health, University College London, London, United Kingdom; 4 Malawi-Liverpool-Wellcome Trust, Blantyre, Malawi

**Keywords:** pneumococcus, PCV13, anti-capsular IgG, BAL

## Abstract

Pneumococcal conjugate vaccine (PCV) efficacy is lower for noninvasive pneumonia than invasive disease. In this study, participants were immunized with 13-valent PCV (PCV13) or hepatitis A vaccine (control). Bronchoalveolar lavage samples were taken between 2 and 6 months and serum at 4 and 7 weeks postvaccination. In the lung, anti-capsular immunoglobulin G (IgG) levels were higher in the PCV13 group compared to controls for all serotypes, except 3 and 6B. Systemically, IgG levels were elevated in the PCV13 group at 4 weeks for all serotypes, except serotype 3. IgG in bronchoalveolar lavage and serum positively correlated for nearly all serotypes. PCV13 shows poor immunogenicity to serotype 3, implying lack of protective efficacy.

**Clinical Trials Registration.** ISRCTN 45340436.

## Teaser

This study found that immunization with 13-valent pneumococcal conjugate vaccine elicits serotype-specific immunoglobulin G (IgG) responses, with serum IgG levels to correlate with those measured in bronchoalveolar lavage samples, but shows poor immunogenicity against serotype 3 in both sites.

Colonization of the human nasopharynx with *Streptococcus pneumoniae* (pneumococcus [Spn]) is a frequent and immunizing event, but local/distal tissue invasion leads to a spectrum of diseases, including pneumonia. Community-acquired pneumonia (CAP) is a leading cause of death across all economic settings [1].

The licensed 7-, 10-, and 13-valent pneumococcal conjugate vaccines (PCV7, PCV10, and PCV13, respectively) are designed to elicit anti-capsular immune responses to some of the most prevalent serotypes causing disease. Currently either PCV10 or PCV13 is included in childhood immunization programs of many countries, whereas a pneumococcal polysaccharide vaccine (PPSV23) is more commonly recommended for older adults and high-risk groups. Despite vaccinations, 2 PCV13-included serotypes, 3 and 19A, have continued to be detected in colonization studies in fully PCV13-vaccinated children [2]. Serotype 3 is especially important in adults, where it makes up a significant amount of remaining pneumococcal disease, the majority of which is CAP [3]. PCV13 efficacy against CAP has been reported to be lower compared to invasive pneumococcal disease (IPD), with a study finding 45% vaccine efficacy for a first episode of vaccine-type nonbacteremic, noninvasive CAP, compared to 75% for IPD [4]. Assessment of the efficacy of PCVs against CAP is more complicated than IPD, due to diagnostic challenges, lower specificity of the endpoint measuring efficacy to CAP, and presence of other respiratory pathogens causing CAP. Estimated lower vaccine efficacy could be the result of reduced humoral responses to PCV13 in the lung mucosa, as failure of local immunoglobulin production in the lung has been associated with increased nosocomial pneumonia in ventilated patients [5]. Herein, we described the anti–capsular polysaccharide (CPS) immunoglobulin G (IgG) responses in the lung lining fluid of healthy adults in response to PCV13 vaccination and compared the levels of anti-CPS IgG to 6B induced by experimental nasal pneumococcal colonization or immunization.

## METHODS

Healthy, nonsmoking participants aged 18–50 years were enrolled into a randomized controlled trial previously described [6]. Ethical approval was given by the Northwest-Liverpool East Research Ethics Committee reference number 12/NW/0873. Participants were randomized to receive the PCV13 (Prevenar-13, Pfizer; n = 49) or hepatitis A (HepA) vaccine (Avaxim, Sanofi Pasteur MSD; n = 50). Serum samples were collected at baseline and at 4 weeks and 7 weeks postvaccination. Five weeks following vaccination, participants were inoculated intranasally with live pneumococcus, serotype 6B, as part of an experimental human pneumococcal challenge study to test efficacy of the vaccine against nasal colonization [7].

A subset (n = 19 [HepA, n = 10 and PCV13, n = 9]) consented to research bronchoscopy, and this subset is the focus of this article (Supplementary Table 1). Bronchoalveolar lavage (BAL) samples were collected at a singular time point between 2 and 6 months postvaccination (Supplementary Figure 1*A*), using a procedure previously described [8]. In addition, a subset of nonvaccinated (PCV13 or PPSV23) participants (n = 63), enrolled in different experimental human pneumococcal challenge studies (15/NW/0146, 14/NW/1460), were stratified based on carriage status to assess the effect of nasopharyngeal pneumococcal colonization on lung mucosa.

IgG levels to capsular polysaccharides of all vaccine serotypes were measured in both serum and BAL samples using the World Health Organization (WHO) standardized enzyme-linked immunosorbent assay (ELISA) method, as previously described [9]. IgG to 6A was not measured due to cross-reactivity with 6B [10]. In brief, ELISA plates were coated with 5 μg/mL of each purified CPS (Statens Serum Institut). BAL supernatant was used undiluted, whereas serum samples were used in three 1:3 serial dilutions, starting from 1:50. Antigen-specific antibodies were detected by goat anti-human IgG (1/4000; Fc-specific) alkaline phosphatase (Sigma). Optical density was measured at 405 nm using FLUOstar Omega plate reader (BMG Labtech).

Multiplex opsonophagocytosis assays were performed in serum samples, using the WHO standard method, as previously described [11]. Three cassettes of pneumococcal bacteria, containing 4 pneumococcal serotypes each resistant to a different antibiotic (Supplementary Table 2) (covering all PCV13 serotypes except serotype 3), were incubated with serial dilutions of human serum, then further incubated with baby rabbit complement and HL-60 cells. Each well was spotted onto 4 blood agar plates and each plate was overlaid with agar containing an antibiotic before overnight incubation, allowing only the correspondingly resistant pneumococcal strain to grow. Opsonophagocytic activity was calculated by determining the concentration of serum at which 50% of bacteria were killed.

### Statistical Analysis

Antibody titers from control and PCV13-immunized subjects were compared using the Mann–Whitney *U* test when 2 groups were compared. Friedman test with Dunn multiple comparisons test was performed when 3 groups were compared. The serum and BAL IgG titers were converted to a log_10_ base and analyzed for correlation with linear regression by the Pearson correlation coefficient using the statistical software R and Prism 8. Significance was set at *P < *.05.

## RESULTS

### PCV13 Elicits Antibody Responses in the Lung Lining Fluid Against All Serotypes, Except Serotype 3

**Figure 1.**
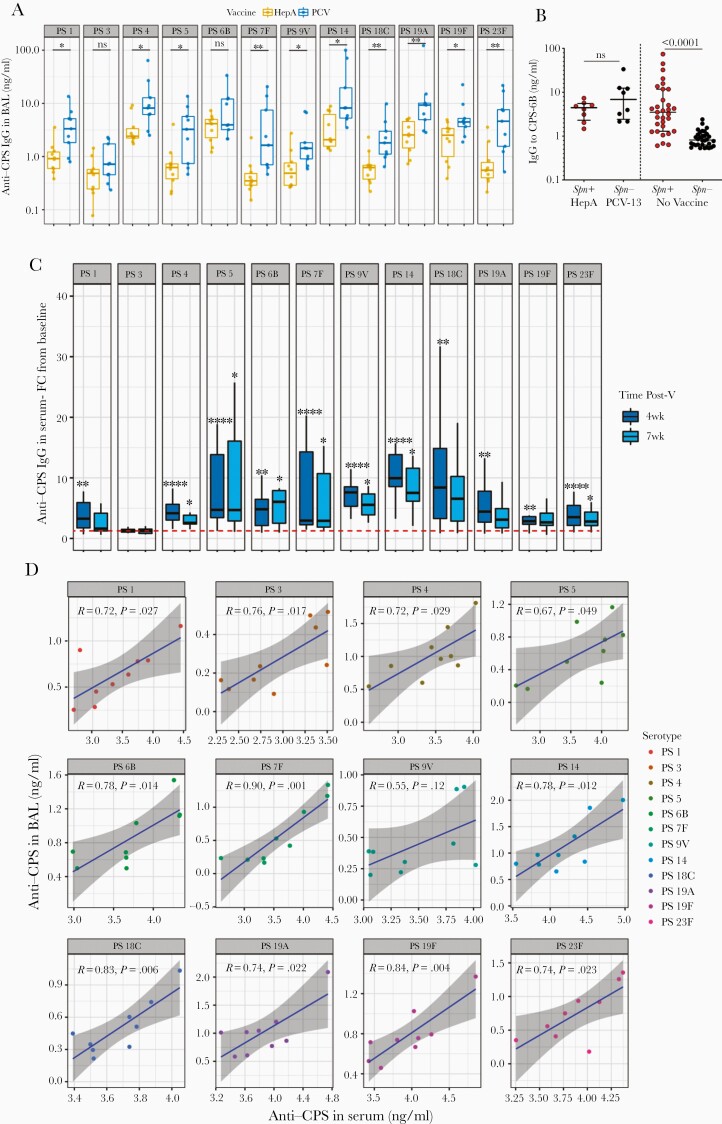
*A*, Levels of immunoglobulin G to capsular polysaccharide (anti-CPS IgG) for 12 pneumococcal serotypes (PS) in bronchoalveolar lavage (BAL) fluid collected between 2 and 6 months postvaccination (Post-V) with either 13-valent pneumococcal conjugate vaccine (PCV13; n = 9) or hepatitis A vaccine (HepA; n = 10). Each dot represents an individual. Error bars depict medians with interquartile range (IQR). Mann–Whitney test was used. *P < .05, **P < .01. *B*, From left to right: Levels of IgG to CPS of *Streptococcus pneumoniae* (Spn) 6B measured in the BAL fluid of HepA/Spn^+^ (n = 8), PCV13/Spn^–^ (n = 8), Spn^+^ (n = 31), and Spn^–^ (n = 32) up to 6 months after pneumococcal challenge. Each dot represents an individual. Error bars depict medians with IQR. *P* value is shown on the graph. Mann–Whitney test was used. *C*, Fold-change (FC) of serum anti-CPS IgG from baseline (prevaccination) at 4 and 7 weeks after vaccination measured against 12 vaccine serotypes. Boxplots depict median and IQR. Dashed line represents FC equal to 1. Friedman test following Dunn multiple comparison test was used. ****P < .0001. *D*, Correlation of anti-CPS IgG levels in serum at 7 weeks after vaccination with the paired anti-CPS IgG levels detected in BAL sample the day of bronchoscopy. Spearman correlation was used and linear regression line with 95% confidence interval (gray shading) is shown.

To investigate whether PCV13 elicits antibody responses in the lung of vaccinated subjects, we used research bronchoscopy to sample the human lung within 6 months after vaccine administration. Levels of anti-CPS IgG in BAL were statistically significantly higher in the PCV13 group (n = 9) compared to the control group (n = 10) for all vaccine antigens, except serotype 3 and 6B (Figure 1A). The highest fold difference in BAL IgG levels between the 2 groups was measured for serotype 23F, whereas the lowest significant difference was measured for serotype 19F (Supplementary Table 2). Anti-CPS IgG to 23F and 19F was, respectively, 8.4- and 1.8-fold higher in the PCV13 group compared to controls (median, 8.4-fold [interquartile range {IQR}, 2.8- to 12.6-fold] and 1.8-fold [IQR, 1.4- to 5.1-fold]). Both HepA and PCV13 subjects were inoculated with Spn 6B at 5 weeks postvaccination, which resulted in 8/10 (80%) and 1/9 (11%) carriage in the HepA and PCV groups, respectively (Supplementary Table 1). Titers of BAL IgG to CPS-6B between experimentally induced pneumococcal carriers (Spn^+^, n = 8) in the control group and noncarriers (Spn^–^, n = 8) in the PCV13 group did not differ significantly (*P* = .32; Figure 1B). This boosting effect of nasopharyngeal pneumococcal colonization in the lung was also observed in a separate nonvaccinated cohort, where Spn carriers (n = 31) had 4.3-fold higher levels of IgG to CPS-6B in the BAL compared to noncarriers (n = 32) up to 6 months postcolonization (median, 3.51 [IQR, 1.19–11.74] in Spn^+^ vs 0.81 [IQR, 0.62–1.11] in Spn^–^) (Figure 1B).

In the analysis of the IgG titers with time, levels of anti-capsular IgG in BAL were sustained at relatively higher levels in PCV13-immunized subjects compared to the controls for up to 4 months postvaccination for most serotypes, although they decreased over time (Supplementary Figure 1*B*). A steeper drop in antibody levels was observed in serotypes that had some of the lowest fold rises compared to the control vaccinated subjects (9V, 19A, 19F) (Supplementary Figure 1*B*).

In blood, baseline levels of anti-CPS IgG increased for all measured vaccine antigens at 4 weeks postvaccination in PCV13 subjects, except serotype 3 (1.25 [IQR, 1.06–1.51] fold-increase; *P* = .129) (Table 1, Figure 1C). Serum anti-CPS IgG to nearly all serotypes, except serotype 3, also increased from baseline to 7 weeks postvaccination. Raised antibody titers decreased from 4 weeks to 7 weeks postvaccination for all the vaccine antigens. This drop was most profound for anti-CPS IgG to 19A, which decreased by 32% over this time period (median, 9.06 [IQR, 3.40–20.02] µg/mL at 4 weeks vs 6.17 [IQR, 3.15–14.10] µg/mL at 7 weeks; *P* = .019). Likewise, in PCV13 subjects, opsonophagocytic killing increased at 4 weeks postvaccination compared to baseline for most vaccine antigens, with killing capacity declining over time (7 weeks postvaccination) (Supplementary Figure 1*C*).

**Table 1.** Systemic Immunoglobulin G to Capsular Polysaccharide for the Vaccine Serotypes

Serotype	PCV13 Fold-Change 4 wk/BL	Adjusted *P* Value	PCV13 Fold-Change 7 wk/BL	Adjusted *P* Value	HepA Fold-Change 4 wk/BL	Adjusted *P* Value	HepA Fold-Change 7 wk/BL	Adjusted *P* Value
1	3.28 (1.76–5.91)	.001	1.63 (1.24–4.16)	.330	0.99 (0.83–1.07)	>.99	0.93 (0.89–0.98)	.38
3	1.25 (1.06–1.51)	.129	1.25 (0.85–1.54)	>.99	0.93 (0.84–1.07)	>.99	0.90 (0.83–1.09)	>.99
4	4.18 (3.05–5.64)	<.0001	2.57 (2.41–3.84)	.023	0.87 (0.80–0.95)	.58	0.82 (0.75–1.0)	.08
5	4.74 (3.46–13.83)	<.0001	4.71 (2.90–16.08)	.012	1.13 (0.93–1.33)	>.99	1.16 (1.01–1.35)	.14
6B	4.94 (3.11–17.51)	.009	6.45 (3.30–12.91)	.017	0.81 (0.76–0.97)	.72	1.62 (1.0–4.18)	.72
7F	2.97 (2.27–14.29)	<.0001	2.92 (1.88–10.70)	.012	0.91 (0.87–1.03)	>.99	1.07 (0.80–1.20)	>.99
9V	7.60 (5.32–8.53)	<.0001	5.55 (3.91–7.35)	.023	0.86 (0.65–1.08)	.87	0.97 (0.61–1.0)	.23
14	9.96 (8.58–13.85)	<.0001	7.53 (6.16–11.59)	.023	0.92 (0.77–0.95)	.38	0.86 (0.71–0.93)	.020
18C	11.46 (3.73–16.17)	.001	8.15 (3.55–11.21)	.076	0.84 (0.81–0.93)	.38	0.86 (0.81–0.98)	.14
19A	4.44 (2.70–7.80)	.003	3.10 (1.76–4.92)	.21	0.81 (0.68–0.89)	.13	0.75 (0.64–0.89)	.075
19F	2.85 (2.35–3.60)	.001	2.67 (2.16–4.18)	.076	0.75 (0.71–0.81	.004	0.81 (0.79–0.90)	.231
23F	3.53 (2.17–5.45)	<.0001	2.81 (2.14–4.36)	.012	1.15 (1.03–1.21	.23	1.17 (0.96–1.43)	.23

Data are presented as median (interquartile range [IQR]) unless otherwise indicated. Table shows the median fold-increase at 4 and 7 weeks postvaccination from baseline and IQR per serotype per vaccinated group (PCV13, n = 9 and HepA, n = 10). For comparison of immunoglobulin G levels between baseline and 4 or 7 weeks, adjusted *P* values are shown as calculated after correction for multiple comparisons using Dunn test.

Abbreviations: BL, baseline; HepA, hepatitis A vaccine; PCV13, 13-valent pneumococcal conjugate vaccine.

Correlation of anti-CPS IgG titers in BAL and paired serum samples at 7 weeks postvaccination in the PCV group demonstrated a positive association for all serotypes, except 9V, with a moderate strength of association for most serotypes (Figure 1D).

## Discussion

This study demonstrates that vaccination with PCV13 elicits IgG responses to vaccine antigens not only systemically but also in the lung mucosa and that pneumococcal colonization with Spn 6B may have a boosting effect in the lung, similar to that induced by intramuscular immunization. Postvaccination, lung IgG levels against the vaccine antigens, although waning over time, were still higher in the PCV13 group for some serotypes compared to control counterparts for up to 4 months. In serum, PCV13 elicited robust serotype-dependent IgG responses. Heightened serum IgG titers began to decline after 4 weeks postvaccination, although they did not differ statistically between 4 and 7 weeks postvaccination. This phenomenon was observed for most measured vaccine serotypes, except serotype 3, in which PCV13 did not induce antibody responses either systemically or in the lung mucosa, implying diminished immunogenicity.

Many studies have reported reduced PCV13 ability to elicit antibody responses against serotype 3 in serum [3] and have suggested that higher IgG concentrations may be required to protect against this serotype [12]. As a result, there is some debate regarding the effectiveness of PCV13 against serotype 3 disease. PCV13’s reduced immunogenicity to serotype 3 may reflect the high circulation of serotype 3 throughout the PCV13 implementation. Clearly, serotype 3 differs from other serotypes, and its ability to evade current vaccine strategies is of concern. On the other hand, PCV13 induced robust IgG responses in blood and lung for serotype 19A. Some possible factors contributing to serotype 19A emergence are high rates of 19A carriage, penicillin nonsusceptibility, and capsular switching [13].

Overall, our blood and BAL findings are in agreement with data from Malawi, whereby vaccination with PCV7, which induces antibody responses to serotypes 4, 6B, 9V, 14, 18C, 19F, and 23F, significantly increased capsular specific IgG to 4 serotypes (6B, 14, 19F, and 23F) in serum and BAL of both human immunodeficiency virus–positive and –negative Malawian adults [5]. In this study, no significant difference was observed between antibody responses to Spn 6B in BAL between PCV13 and control-vaccinated subjects, as both groups were challenged with 6B at 5 weeks after vaccination. The pneumococcal challenge resulted in disproportionally higher pneumococcal colonization rates in the control group (80% in control vs 11% in the PCV group) and elicited similar levels of lung antibodies to CPS of 6B in the colonized, control vaccinees compared to noncolonized, PCV13 vaccinees. As opposed to a study of inhaled delivery of PPSV23 [14], nasopharyngeal pneumococcal colonization induced mucosal immune responses in the lung of colonized subjects. This is consistent with previous work from our group, which has shown that human pneumococcal colonization has an immunogenic effect in the lung by seeding the lung mucosa with pneumococcal-specific CD4^+^ T cells [15]. In this study, cellular responses, such as tissue resident polysaccharide-specific B cells, following PCV13 vaccination were not measured. However, as memory B cells are an important aspect of lung immune defenses, future studies are needed to focus on the role of resident B cells in the lung in response to vaccination and the local IgG production—knowledge that will contribute to better understand the relationship of PCVs and the lower vaccine efficacy witnessed against CAP compared to IPD.

Some of the limitations of this study are the small sample size and the difference in timing of sample collection between serum and BAL samples, which may have affected the strength of correlation between these 2 sites. Despite this, the study shows an appropriate response to PCV13 in lung fluid up to 4 months postvaccination. Also, the positive association between systemic and lung IgG suggests antibody diffusion from blood to mucosa, providing assistance to the site of infection. These findings work against the notion that poor humoral responses at the lung mucosa play a role in reduced vaccine efficacy for most serotypes, except serotype 3. Our findings imply that discrepancies in vaccine efficacy against pneumonia could be due to several other factors, such as viral coinfection causing CAP, diminished lung resident cell function during such coinfections, or challenges to ascertain pneumonia etiology. On the other hand, lack of immunogenicity against serotype 3 supports numerous data on reduced vaccine efficacy for this serotype. Additionally, the study encourages the idea of developing a live attenuated pneumococcal vaccine for inhaled delivery for the prevention of pneumonia.

## Supplementary Data

Supplementary materials are available at *The Journal of Infectious Diseases* online. Consisting of data provided by the authors to benefit the reader, the posted materials are not copyedited and are the sole responsibility of the authors, so questions or comments should be addressed to the corresponding author.

jiab331_suppl_Supplementary_Material

## Notes

***Author contributions.*** E. M. and D. M. F. conceived and designed the study. E. M., D. M., A.-S. W., and S. J. analyzed and interpreted the data. A. H.-W. and A. M. C. assisted in clinical procedures and recruitment. D. M. wrote the first draft of the manuscript. E. M., A.-S. W., D. G., R. S. H., S. B. G., A. M. C., and D. M. F. commented on and approved the manuscript.

***Acknowledgments.*** The authors thank all the volunteers for their participation in the clinical trial and all the clinical team members for helping to collect the samples. The authors also thank the team at Liverpool University Hospitals NHS Foundation Trust for their assistance, particularly the Clinical Research Unit at the Royal Liverpool, as well as the National Institute for Health Research (NIHR) Clinical Research Network in the North West Coast.

***Financial support.*** This work was supported by the Bill & Melinda Gates Foundation (grant number OPP1117728 to D. M. F.) and the Medical Research Council (grant number MR/M011569/1 to S. B. G.). R. S. H., A.-S. W., and D. G. are supported by the NIHR Global Health Research Unit on Mucosal Pathogens using aid from the UK government.

***Potential conflicts of interest.*** All authors: No reported conflicts of interest.
