# Supplementary material for: Thirteen-Valent Pneumococcal Conjugate Vaccine–Induced Immunoglobulin G (IgG) Responses in Serum Associated With Serotype-Specific IgG in the Lung
Source: J Infect Dis. 2021 Jun 22;225(9):1626–31. doi: 10.1093/infdis/jiab331 (PMC9071286; doi:10.1093/infdis/jiab331)
Supplement: jiab331_suppl_Supplementary_Material [file jiab331_suppl_supplementary_material.docx]

**A**


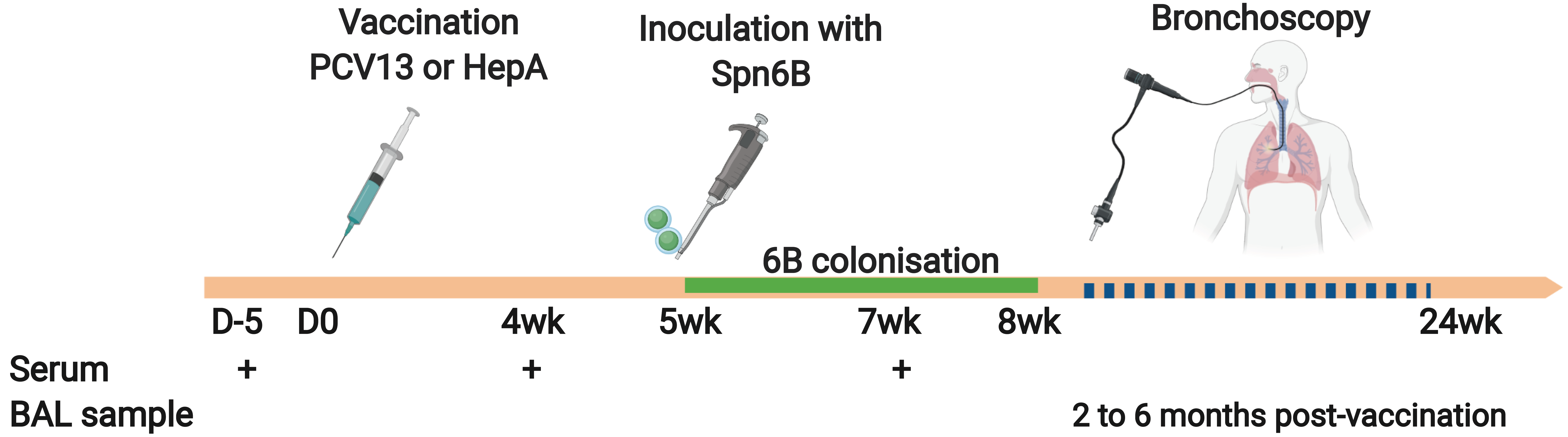


**B**

**C**
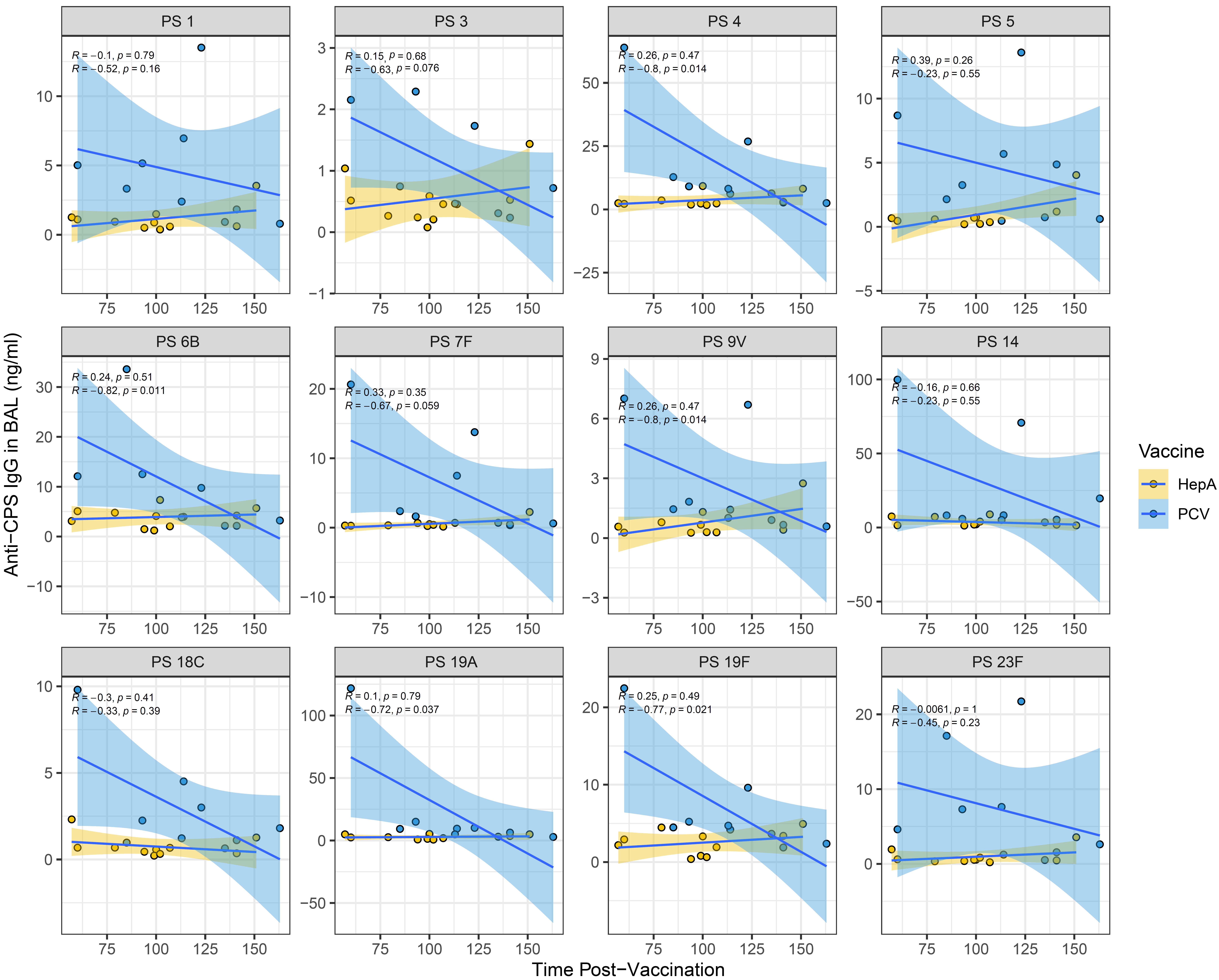


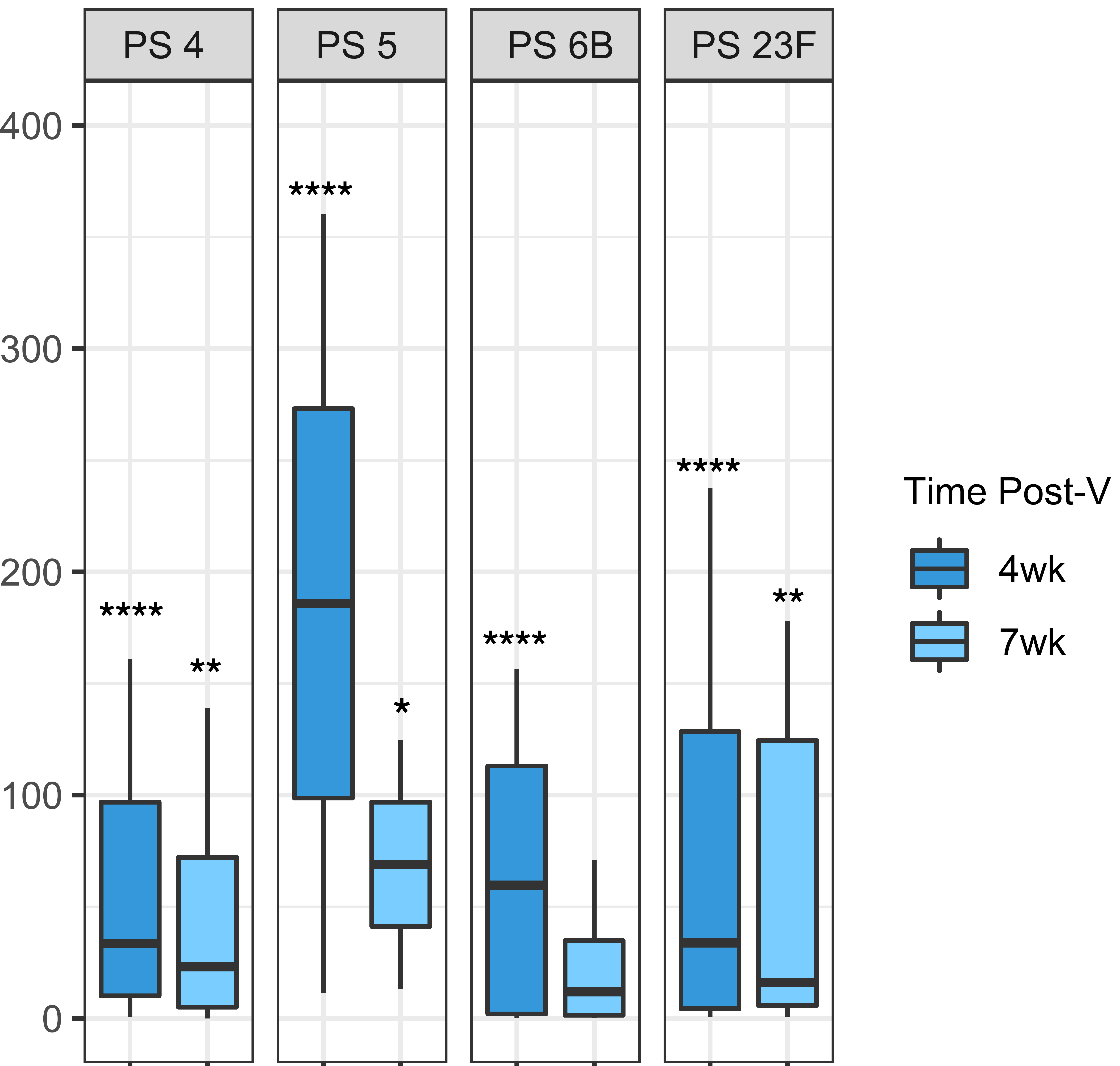

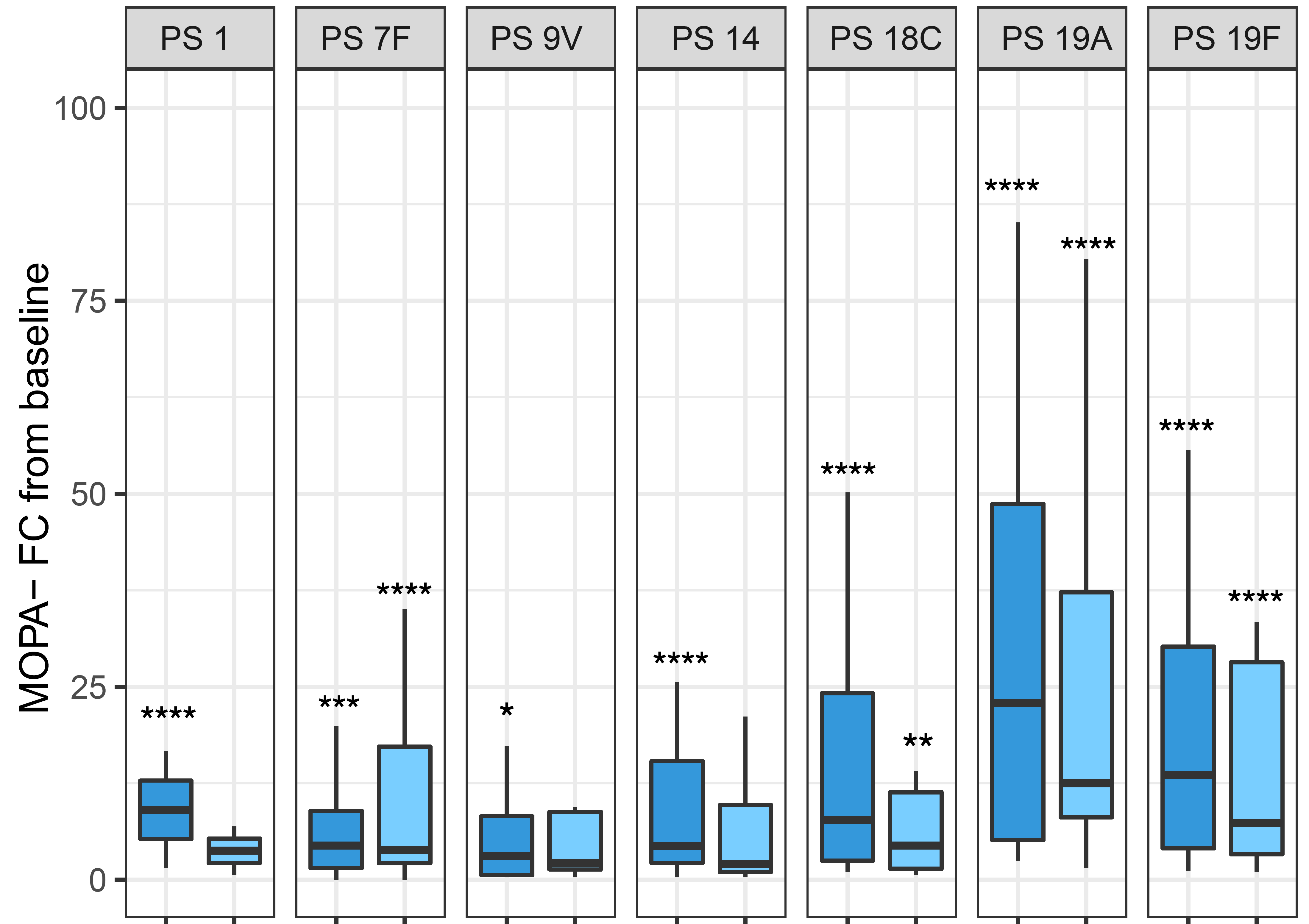


**Figure S1.** **A)** Study design. Baseline serum was collected 5 days before vaccination (D-5 with either HepA or PCV13 vaccine (D0). Serum samples were collected at 4- and 7-weeks post vaccination (PV). All participants were inoculated with live 6B *Streptococcus pneumoniae* 5 weeks after vaccination. Bronchoalveolar lavage (BAL) samples (n=19) were collected at a singular time point between 2 and 6 months after vaccination. **B)** Continuous linear model (lines) and observed titers (dots) of IgG to capsular polysaccharide of 12 vaccine-serotypes over time. Association between the two ranked variables per group (HepA in yellow and PCV in blue) per serotype was tested using Spearman correlation test. R and p values are shown. 95% confidence interval (shedding) are shown. **C)** Fold change of serum OPA from baseline (pre-vaccination) at 4- and 7-weeks post vaccination measused against 11 vaccine-serotypes (PCV vaccinated, n=19). Boxplots dipict median and IQR. Friedman test, following Dunn’s multiple comparison test was used.

**Table S1: Demographics of study participants** Includes data from PCV/EHPC study (Collins et al., 2015).

| **PCV/EHPC- BAL subset** | **Hep A** | **PCV13** |
| --- | --- | --- |
| No. of subjects | 10 | 9 |
| Age (mean ± SD) | 24.6 ± 9.8 | 25.7 ± 6.0 |
| Sex (M: F) | 2:8 | 6:3 |
| Median time of BAL post-Vaccination (range) in days | 99.5 (57-151) | 114.0 (60-163) |
| Carriage rate | 80% (8/10) | 11% (1/9) |

Abbreviations: No.= number, SD= standard deviation, BAL= bronchoalveolar lavage, Hep A= hepatitis A, M= male, F= female

**Table S2: Pneumococcal serotypes used in the study.** Serotype 6B (BHN418) used in intranasal challenge is a clinical isolate from paediatric otitis media. All strains used in MOPA assays have been obtained from BEI Resources.

| **Pneumococcal serotypes** | **Strain** | **Antibiotic Resistance** | **Source** |
| --- | --- | --- | --- |
| Serotype 6B (Challenge strain) | BHN418 | Not resistant to antibiotics | Clinical isolate |
| Serotype 1 | SPEC1 | Spectinomycin resistant | BEI/ NR-13388 |
| Serotype 4 | OREP4 | Optochin resistant | BEI/ NR-13390 |
| Serotype 5 | STREP5 | Steptomycin resistant | BEI/ NR-13391 |
| Serotype 6A | TREP6A | Trimethoprin resistant | BEI/ NR-13392 |
| Serotype 6B | SPEC6B | Spectinomycin resistant | BEI/ NR-13393 |
| Serotype 7F | OREP7F | Optochin resistant | BEI/ NR-13394 |
| Serotype 9V | EMC9V | Streptomycin resistant | BEI/ NR-13395 |
| Serotype 14 | STREP14 | Steptomycin resistant | BEI/ NR-13396 |
| Serotype 18C | OREP18C | Optochin resistant | BEI/ NR-13397 |
| Serotype 19A | TREP19A | Trimethoprin resistant | BEI/ NR-13398 |
| Serotype 19F | SPEC19F | Spectinomycin resistant | BEI/ NR-13399 |
| Serotype 23F | EMC23F | Clinical isolate (1212458), naturally resistant to trimethoprim | BEI/ NR-13400 |

BEI: NIH Biodefense and Emerging Infections Research Resources Repository, NIAID, NIH
